# Regulatory Acceptance of Patient-Reported Outcome (PRO) Data from Bring-Your-Own-Device (BYOD) Solutions to Support Medical Product Labeling Claims: Let’s Share the Success Stories to Move the Industry Forward

**DOI:** 10.1007/s43441-022-00412-1

**Published:** 2022-05-09

**Authors:** Florence D. Mowlem, Pamela Tenaerts, Chad Gwaltney, Ingrid Oakley-Girvan

**Affiliations:** 1 Medable Inc, Palo Alto, CA USA; 2 Gwaltney Consulting, Westerly, RI USA

**Keywords:** Bring-your-own-device (BYOD), Patient-reported outcome (PRO), Electronic clinical outcome assessment (eCOA), Decentralized trial (DCT), Labeling claim

## Abstract

Bring-your-own-device (BYOD) methods for collecting patient-reported outcome (PRO) data in clinical trials can decrease patient burden and improve data quality. However, adoption of BYOD in clinical trials is limited by the absence of publicly available case studies where BYOD PRO data supported regulatory medical product approvals. Anecdotally, we are aware of multiple examples where efficacy and safety label claims were based on BYOD PRO data; however—except for one—these examples have not been made public. The absence of these case studies can lead sponsors to be hesitant to use BYOD for capturing primary and secondary PRO-based endpoints in their trials. This commentary outlines the context of the issue faced and concludes with a call for sponsor transparency with regard to BYOD use through publicizing where approved labeling claims were based on BYOD data. We suggest how this data could be systematically captured going forward. Sharing this information will benefit the clinical trials enterprise by increasing confidence in the utilization of BYOD and provide opportunities to enhance patient-centricity.

Data from patient-reported outcome (PRO) measures are now routinely used as endpoints in clinical trials to elicit the patient perspective [1] and drive greater incorporation of the patient voice in medical product development. For example, between 2016 and 2020, 26.3% of new drug applications (NDAs) reviewed by the US Food and Drug Administration (FDA) had labeling statements based on PRO endpoints, and 50% of NDAs for diseases that traditionally rely on PRO assessments to derive or construct the primary or secondary endpoints for the evaluation of treatment benefit by regulators, had PRO labeling statements [2]. The importance of including the patient perspective in drug development is detailed in a series of recent guidances from the FDA that outline methods for “Enhancing the Incorporation of the Patient’s Voice in Medical Product Development and Regulatory Decision Making”, with the aim of ensuring greater patient focus in medical product development and meet the requirements of the 21st Century Cures Act of 2016 and Prescription Drug User Fee Act (PDUFA) VI [3, 4].

PROs can be collected at the research site or outside of the clinic environment [5]. Remote PRO data capture can facilitate real-time understanding of a treatment’s safety and effectiveness by measuring symptoms and functional impacts in the participant’s usual environment instead of solely within a clinical setting [6]. Within the clinical trials industry, administering PRO instruments electronically (electronic patient-reported outcomes/ePRO) has become commonplace [7–9], especially when collecting data remotely at defined time points as it enables assurance of the contemporaneousness of the data. In addition, remote data capture can reduce both site and participant burden by enabling reduced frequency and duration of face-to-face clinic visits [3]; thus, offering the opportunity to facilitate increased adoption of the decentralized trial (DCT) model and enhance patient-centricity in clinical trials.

Historically, participants have been provisioned devices solely for the completion of ePROs. However, in recent years there has been increased interest in taking advantage of the option for participants to use their own device (smartphone, tablet, laptop) for completing study questionnaires—known as ‘bring-your-own-device’ (BYOD) [7].

Studies have shown that many individuals may prefer to use their own device to respond to PRO instruments [10–12], and so the option of BYOD to capture PRO data can help address patient-centricity by improving the ease of participation. As participants are familiar with their own devices, BYOD could increase engagement and compliance, potentially improving retention and reducing missing data [8]. Missing PRO data have been identified as one of the main problems for the interpretation of PRO-based endpoints and their subsequent use in the labeling of medical products [13–15].

Greater ease of use and familiarity with their own device may be especially pertinent in certain therapeutic areas where the condition and/or side-effects of treatment may affect an individual’s ability to interact with certain electronic devices (e.g., individuals with Parkinson’s Disease experiencing dexterity difficulties or individuals with visual impairment may have difficulties using devices with certain screen sizes). It is unlikely that an individual would own a device that they are not able to use comfortably, and yet this could be the case with a provisioned device. Given the diversity of options (e.g., use of the participant’s own smartphone, tablet, laptop, computer, or trial provisioned devices) now available, we can and should be more responsive to the patient population’s unique needs.

It is important to note that when adopting a BYOD strategy in a clinical trial, it is recommended that sponsors also plan to have a percentage of provisioned devices available for use by study participants to ensure that participants who do not wish to use their own device or do not have a suitable device are still able to take part (and are not excluded from participating, creating bias in study findings) [7]. Furthermore, it is important to recognize that some participants may have concerns around data privacy and security associated with using their own device to submit health data [9], and thus may prefer to use a provisioned device. That said, despite the importance of due consideration to data privacy and security, the trial ePRO software should always have the correct level of security controls built in, regardless of whether the software will be used on a provisioned device or BYOD. Ultimately, being flexible in our approach to data collection modes in clinical trials is likely to have the most beneficial effect for data quality and enhance patient-centricity.

However, utilization of BYOD solutions in clinical trials to capture endpoints intended for use in a labeling claim has been met with heightened uncertainty from drug developers. Sponsors may be reluctant to incorporate BYOD in clinical trials in part due to, (1) concerns and uncertainties about establishing the reliability and validity of remote ePRO data collected using BYOD instead of provisioned devices [5, 8]; (2) the lack of regulatory guidance regarding BYOD use in clinical trials; and (3) the dearth of publicly available case studies where PRO-based endpoints collected using BYOD supported product approval and/or claims in the product labeling.

Fortunately, we are now at a point as an industry where the evidence base, including multiple meta-analyses, has established that electronic versions of PRO instruments are comparable to paper versions (where comprised of standard item and response scales types, modifications made during migration are not substantial, and ePRO design best practices have been followed) [7, 8, 11, 16]. Furthermore, studies specifically comparing data collected with BYOD to provisioned devices demonstrate equivalence, and this growing body of evidence supports the reliability and validity of PROs measured using BYOD solutions [7, 8, 11, 17, 18]. Thus, we hope that this can mitigate the concerns many sponsors still have around measurement equivalence of BYOD with paper and provisioned devices.

It is also known that a regulatory approved labeling claim based on ePRO data collected using BYOD *has* occurred, which should instill confidence. The Pfizer and BioNTech mRNA Vaccine study for the treatment of COVID-19 collected PRO safety data from over 40,000 participants worldwide using an electronic diary, with 79% of participants using their own device (BYOD) and 29% using a provisioned device [19]. Given BYOD solutions have been used for many years with increasing adoption in clinical trials, it is reasonable to assume that there are more instances of BYOD data successfully being submitted to regulatory agencies for use in a medical product labeling claim; indeed, anecdotally this has been reported. The problem is, in the ecosystem of clinical trials, anecdotes are not enough. We need publicly available case studies where BYOD data have been used to support regulatory approval and product labeling. Having this evidence is likely to increase adoption, as well as confidence in the use of BYOD solutions to capture PRO measures in clinical trials, where such data forms primary and/or secondary endpoints.

Currently, documenting these further examples poses a challenge. Regulatory review documentation for new product approvals often does not indicate if PRO data were collected using BYOD. Documentation may specify that data was captured electronically but not whether the device was BYOD or provisioned. A detailed review of trials registered on Clinicaltrials.gov would also not provide information on provisioned or BYOD methodology, as trial sponsors are only required to disclose the measurement and not the modes of administration used to capture that data. Electronic clinical outcome assessment (eCOA) vendors who provide BYOD solutions for capturing ePRO data often lack the insight as to whether the submission was subsequently approved by the regulatory agency and the PRO data used on the label. Extracting this knowledge requires a manual and time-consuming process of linking studies the vendor supported with BYOD, to reviews and labels for those medical products. The ability of vendors to document and publicize this type of information may be further limited by confidentiality agreements.

Due to the absence of a central resource where the use of BYOD in clinical trials is systematically documented, an approach to transparently communicating this information is needed. To facilitate dissemination of this information, we suggest a neutral, non-commercial entity be the host of a suitable repository, such as the Critical Path Institute’s (C-Path) eCOA Consortium, Drug Information Association’s (DIA) Study Endpoints Group, the International Society for Patient Outcome Research (ISPOR), or another industry consortium with a focus on COA measures and endpoints. We envisage that such a repository could function in a similar way as the Digital Medicine Society’s (DiME) crowdsourced library of digital endpoints, where pharma companies have publicly disclosed that they have collected digital endpoints in their trial and provided details around these, and access to this database is openly available to all [20].

Table 1 provides an example of the information that could be included in this library, using the Pfizer-BioNTech COVID-19 vaccine for demonstration purposes. This living resource, where sponsors can transparently disclose they have received approved PRO labeling claims using BYOD, would represent the commitment of the drug development industry to patient-centric approaches in clinical research.

**Table 1:** Proposed data to capture in a library of studies utilizing BYOD to capture PRO safety or efficacy endpoints that were used on the label of approved medical products

Sponsor	TI	Product name	Study phase	Endpoint position and Type	PROM	PRO endpoint	% of BYOD	ClinicalTrials.gov identifier	Approval agencies	PI link
BioNTech SE, Pfizer	COVID-19	COMIRNATY	Phase 3	Primary safety	Solicited local and systemic reaction diary	% of participants reporting the following for 7 days after Dose1, 2, and 3: pain, redness, and swelling at injection site, fatigue, headache, muscle pain, chills, joint pain, fever, vomiting, diarrhea	79%	NCT04713553	FDA, EMA	https://www.fda.gov/media/151707/download

*TI* therapeutic indication, *PROM* patient reported outcome measure, *BYOD* bring your own device, *PI* package insert

Thus, in an era of increasing pressure for transparency in clinical research to progress the field and embody patient-centricity, *we are asking sponsors with approved efficacy- or safety-related labeling claims based on BYOD (fully or partially) captured PRO data to share this success with the industry.* We strongly believe this initiative holds the potential to overcome some key concerns from sponsors around the use of BYOD to collect PRO data that will be submitted to a regulatory agency and used in medical product labeling, and increase the thoughtful adoption of BYOD in clinical trials.

For the purposes of this commentary, we have focused on BYOD PRO data that have been used for labeling claims as we see this as the most pressing need. However, we do not believe that such a database would need to be restricted to this and can capture evidence of any registrational trials incorporating BYOD strategies for PRO data capture.

Sharing this information will provide benefits to the clinical trials enterprise and patients alike. With the clinical trials and drug development industries evolving at an accelerated pace to deploy and increase adoption of patient-centric approaches, including decentralized technologies, the time is right to resolve this issue, share the success stories, and enhance everyone’s knowledge with such an initiative.
